# Patient-derived multicellular tumor spheroids towards optimized treatment for patients with hepatocellular carcinoma

**DOI:** 10.1186/s13046-018-0752-0

**Published:** 2018-05-25

**Authors:** Yeonhwa Song, Jin-Sun Kim, Se-Hyuk Kim, Yoon Kyung Park, Eunsil Yu, Ki-Hun Kim, Eul-Ju Seo, Heung-Bum Oh, Han Chu Lee, Kang Mo Kim, Haeng Ran Seo

**Affiliations:** 1 Cancer Biology Research Laboratory, Institut Pasteur Korea, 16, Daewangpangyo-ro 712 beon-gil, Bundang-gu, Seongnam-si, Gyeonggi-do 13488 Korea; 2 Division of Gastroenterology and Hepatology, ASAN Medical center, Olympic-ro 43-gil, Songpa-gu, Seoul 05505 Korea; 3 Department of Pathology, University of Ulsan College of Medicine, Seoul, 05505 Korea; 4 Division of Hepatobiliary Surgery and Liver Transplantation, Department of Surgery, University of Ulsan College of Medicine, Seoul, 05505 Korea; 5 Department of Laboratory Medicine, Asan Medical, Center, University of Ulsan College of Medicine, Seoul, 05505 Korea

**Keywords:** Hepatocellular carcinoma (HCC), Multicellular tumor spheroids (MCTS), Optimized treatment, MCTS-based chemosensitivity assays

## Abstract

**Background:**

Hepatocellular carcinoma (HCC) is one of the most common malignant tumors worldwide and has poor prognosis. Specially, patients with HCC usually have poor tolerance of systemic chemotherapy, because HCCs develop from chronically damaged tissue that contains considerable inflammation, fibrosis, and cirrhosis. Since HCC exhibits highly heterogeneous molecular characteristics, a proper in vitro system is required for the study of HCC pathogenesis. To this end, we have established two new hepatitis B virus (HBV) DNA-secreting HCC cell lines from infected patients.

**Methods:**

Based on these two new HCC cell lines, we have developed chemosensitivity assays for patient-derived multicellular tumor spheroids (MCTSs) in order to select optimized anti-cancer drugs to provide more informative data for clinical drug application. To monitor the effect of the interaction of cancer cells and stromal cells in MCTS, we used a 3D co-culture model with patient-derived HCC cells and stromal cells from human hepatic stellate cells, human fibroblasts, and human umbilical vein endothelial cells to facilitate screening for optimized cancer therapy.

**Results:**

To validate our system, we performed a comparison of chemosensitivity of the three culture systems, which are monolayer culture system, tumor spheroids, and MCTSs of patient-derived cells, to sorafenib, 5-fluorouracil, and cisplatin, as these compounds are typically standard therapy for advanced HCC in South Korea.

**Conclusion:**

In summary, these findings suggest that the MCTS culture system is the best methodology for screening for optimized treatment for each patients with HCC, because tumor spheroids not only mirror the 3D cellular context of the tumors but also exhibit therapeutically relevant pathophysiological gradients and heterogeneity of in vivo tumors.

**Electronic supplementary material:**

The online version of this article (10.1186/s13046-018-0752-0) contains supplementary material, which is available to authorized users.

## Background

Hepatocellular carcinoma (HCC) is one of the most common malignant tumors worldwide and the third leading cause of cancer mortality [1, 2]. The genetic and physiological complexity of HCC is a major obstacle to the study of tumorigenesis and identification of therapeutic targets. HCC patients typically have poor tolerance of systemic chemotherapy due to underlying liver dysfunction and are resistant to conventional chemotherapeutic agents. Nevertheless, effective chemotherapy and other treatment strategies for patients with HCC are still lacking. Clinicians attempt to select the most effective anti-cancer treatment in order to improve the quality of lifer of HCC patients. To this end, personalized medicine, the potential to tailor therapy with the best response and highest safety margin to ensure better patient care, is optimal to reduce unnecessary exposure to chemotherapy, which has limited efficacy and/or acute toxicity in HCC patients. However, the development of “personalized” treatments for HCC patients is plagued by limitations, such as a lack of fresh biopsy from advanced HCC, the low incidence of genetic driver mutations in HCC, and the tumor heterogeneity. Therefore, suitable experimental models are needed to overcome these problems and to explore the mechanism of HCC development, progression, and metastasis. In this study, we established and characterized two new HCC cell lines, AMC-H1 and AMC-H2, from HBV-infected HCC patients as experimental model of personalized medicine. Both cell lines displayed common hepatocytes characteristics but differed in other way from each other. Also, these secreted HBV DNA stably after serial culture for more than 100 passages.

The 3D culture models have widely been used for the past two decades in an effort to mimic the in vivo behavior of normal or transformed cells under conditions more amenable to experimental investigation. Currently, these methods have been routinely applied to the analysis of malignant transformation and tumor progression. These 3D cultures have potential to greatly improve cell-based drug screening and identify toxic and ineffective substances at an earlier stage of the drug discovery pipeline than animal or clinical trials. Additionally, spheroids composed of patient-derived tissues maintained their own characteristics, such as high levels of glucose consumption, lactate production, and HIF1a levels as well as lower levels of radiation-induced oxidative stress, for a long time in vitro, and studies have suggested that these models serve as proper culture methods for personalized therapy in colorectal cancer [3], glioma [4], and head and neck cancer [5]. Moreover, a 3D system with homogenous and automated methods in the extracellular matrix (ECM) was developed and used to visualize and analyze the migration and invasion ability of cancer cells using mouse breast and human sarcoma biopsy samples [6] as well as melanoma cells [7]. In our previous study, we probed the crosstalk between HCC and tumor microenvironments (TME) that promoted HCC chemoresistance and migration in multicellular tumor spheroids (MCTS). Our results suggested that targeting TME components may offer a promising therapeutic strategy for HCC liver cancer therapy. Thus, in this study, we propose to apply the 3D co-culture model to a system of patient-derived HCC cells and stromal cells representing human hepatic stellate cells (HSCs), human fibroblasts, and human umbilical vein endothelial cells (HUVECs), to screen for personalized cancer therapy. Ultimately, we aim to provide more informative results for clinical drug application through convergence of patient-derived liver cancer cells and TME components in MCTS.

## Methods

### Cell lines and culture conditions

The stromal cells WI38 (human fibroblasts), LX2 (human hepatic stellate cells), and HUVECs (human umbilical vein endothelial cells) were purchased from ATCC (Manassas, VA, USA), Merck Millipore, (Darmstadt, Germany), and PromoCells (Heidelberg, Germany), respectively. All cells were maintained at 37 °C in a humidified atmosphere of 5% CO_2_.

WI38 cells were cultured in Minimum Essential Media (MEM; Welgene, Daegu, Korea) supplemented with 10% heat-inactivated fetal bovine serum (FBS; Gibco, Grand Island, NY, USA) and 1× penicillin-streptomycin (P/S; Gibco). LX2 cells were cultured in Dulbecco’s Modified Eagle’s Medium (DMEM; Welgene) supplemented with 2% heat-inactivated FBS and 1× P/S. HUVECs were cultured in endothelial basal medium (EBM) purchased from PromoCells.

### Primary culture of HCCs

Immediately after surgery, a portion of the excised tumor was immersed in Hanks balanced salt solution (HBSS; Gibco) and transported from the operating room at 0 °C to the laboratory. The specimens were collected under sterile conditions and rinsed 2-3 times with HBSS free of calcium and magnesium to remove blood. After removal of blood, the liver sample was cut into small fragments, gently dispersed, and placed in HBSS containing 0.03% pronase (Gibco), 0.05% type IV collagenase (Gibco), and 0.01% deoxyribonuclease (DNase, from bovine pancreas, Gibco) for 20 min at 37 °C. The resultant suspension was filtered through a 100-μm-nylon filter (BD Falcon, Franklin Lakes, NJ, USA) and centrifuged at 50 x *g* for 2 min at 4 °C to obtain hepatocytes. The pellet was washed twice in HBSS containing 0.005% DNase. The final cell suspensions were cultured in collagen-coated T25 flasks (BD Falcon) in hepatocyte basal medium (Lonza, Basel, Switzerland) supplemented with 10% heat-inactivated FBS, 1 ng/ml hepatocyte growth factor (HGF, Prospec, Rehovot, Israel), and 1× antibiotic-antimycotic (Gibco) as HBM media at 37 °C in a humidified incubator with 5% CO_2_. The medium was changed 24 h after seeding to remove dead cells and debris. When cells reached 70-80% confluence, the cells were re-plated in HBM medium with supplements. Confluent cells were trypsinized, counted, and diluted 1:3-1:5 at every passage. Once cell lines were maintained for more than 30 passages, the cells were collected and stored in liquid nitrogen.

### Ethics approval and consent to participate

The study was conducted in accordance with the Declaration of Helsinki principles. The study was approved by the Human Research Ethics Committee of ASAN Medical Center (Permit Number: 2007-0332). The institutional review board at ASAN Medical Center complies with all applicable guidelines, including the ICH, KGCP, and bioethics and safety act. Written informed consent for the use of tissues for research was obtained from patients at the time of procurement of tumor specimens. One line named AMC-H1 was acquired from a 55-year-old female patient, and another, AMC-H2, was from a 51-year-old male patient. The etiology of HCC was HBV infection in both patients.

### Immunocytochemistry

To validate the primary cells, cells were fixed with 4% paraformaldehyde (PFA; Sigma, St Louis, MO, USA) for 10 min at room temperature, permeabilized with 0.1% Triton X-100 (Sigma) in Dulbecco’s phosphate-buffered saline (DPBS; Welgene) for 30 min at room temperature, and then washed three times with DPBS. The following primary antibodies were used: mouse monoclonal anti-human serum albumin (ALB; 15C7, ab10241, 1:500; Abcam, Cambridge, UK), mouse monoclonal anti-human Hep Par-1 (Clone OCH1E5, M7158, 1:100; Agilent Technologies, Santa Clara, CA, USA), and mouse monoclonal anti-human CD133/1 (AC133, 130-090-422, 1:100; Miltenyi Biotec, Bergisch Gladbach, Germany). Samples were incubated with the primary antibodies for 16 h at 4 °C and then washed for 10 min three times with DPBS. The secondary antibodies used for staining were goat anti-mouse IgG conjugated with Alexa® Fluor 488 and goat anti-rabbit IgG conjugated with Alexa® Fluor 488 (Invitrogen, Eugene, OR, USA). Samples were then incubated with secondary antibodies for 1 h at room temperature in the dark and washed for 10 min five times with DPBS. For nuclei staining, cells were incubated with Hoechst 33,342 (Invitrogen) for 10 min at room temperature in the dark and washed with PBS twice quickly. All fluorescence images were obtained using the Operetta® High Content Screening (HCS) System (Perkin Elmer, Waltham, MA, USA).

### Growth properties of AMC-H1 and AMC-H2

The AMC-H1 and AMC-H2 cell lines were seeded at 1 × 10^3^ cells per well in 96-well plates and allowed to grow 1-7 d. Cell viability was assessed by the MTS assay (Promega, Madison, WI, USA) at the indicated times.

### LCSC spheroid formation

AMC-H1 and AMC-H2 primary cells were seeded in 6-well ultra-low attachment (ULA) plates (Corning Life Sciences, Amsterdam, The Netherlands) in DMEM/F12 (Gibco) supplemented with 1× B27 (Invitrogen), 20 ng/ml basic fibroblast growth factor (bFGF; Invitrogen), 20 ng/ml epidermal growth factor (EGF; Invitrogen), 25 μg/ml insulin (Sigma-Aldrich) as LCSC media. Cells were cultivated for 5-7 d without changing the LCSC medium.

### Wound healing assay

To examine metastatic potential, a wound healing assay was performed. Cells were plated in a 6-well plate and grown to 80-90% confluence. After reaching the appropriate confluence, a scratch to generate a wound edge was made through the monolayer using a pipette tip. The migration of cells from the edge of the wound was monitored. Time-lapse images were captured using a microscope equipped with a Nikon 3 camera.

### Western blot analysis

Molecular profiles were examined by western blot analysis. Cells were collected and centrifuged at 1000 rpm for 3 min. The cell pellets were collected and lysed using RIPA buffer (InTron, Jungwon-gu, South Korea) for 30 min at 4 °C with vigorous vortexing. The resultant lysate was centrifuged at 12,500 rpm for 20 min at 4 °C, and the supernatants were collected. The protein concentration was measured with the BCA assay (Promega). Following SDS-PAGE and transfer, membranes were blotted with antibodies to the epidermal growth factor receptor (EGFR, Cell Signaling, Danvers, MA, USA), β-catenin (Cell Signaling), N-cadherin (Cell Signaling), p53 (Santa Cruz Biotechnology, Dallas, TX, USA), PTEN (Cell Signaling), pERK (Cell Signaling), ERK (Cell Signaling), pAkt (Cell Signaling), and Akt (Cell Signaling). Following binding to the appropriate secondary antibodies, signals were detected using ECL reagents (GE, Fairfield, CT, USA).

### Single nucleotide polymorphism (SNP) microarray

To examine the genomic aberrations in AMC-H1 and AMC-H2 cells, we performed SNP array analysis using Affymetrix CytoScanTM 750 K (Affymetrix, Santa Clara, CA, USA). DNA was isolated from AMC-H1 and AMC-H2 using the AxyPrep Multisource Genomic DNA Kit (Axygen, Union City, CA, USA). The SNP microarray was performed according to the standardized protocol provided by the manufacturer. The raw data were analyzed using the Chromosome Analysis Suite 2.0 software (Affymetrix) to detect copy number (CN) alterations and loss of heterozygosity (LOH).

### Tumor spheroid and multicellular tumor spheroid (MCTS) formation and drug treatment

To generate tumor spheroids, cells suspended in HBM media were seeded at a density of 6 × 10^3^ cells/well in 96-well round bottom ULA microplates (Corning Life Sciences). The plates were incubated for 3 d at 37 °C in a humidified atmosphere of 5% CO_2_. To produce MCTSs, four kinds of cells (primary HCC cells, LX2 cells, WI38 cells, and HUVECs) suspended in HBM media were seeded at a density of 6 × 10^3^ cells/well in 96-well round bottom ULA microplates. The plates were incubated for 3 d at 37 °C in a humidified atmosphere of 5% CO_2_. After 3 d, 5-fluorouracil (5-FU; Sigma-Aldrich), cisplatin (Sigma-Aldrich), and sorafenib (Santa Cruz Biotechnology) were added and incubated for an additional 7 d. A solution of 0.5% dimethylsulfoxide (DMSO; Sigma-Aldrich) was used as a negative control.

### Drug sensitivity in monolayer culture conditions

Primary HCC cells were seeded at a density of 2.5 × 10^3^ cells/well in 384-well plates (Greiner Bio-One, Monroe, NC, USA). After a 16-h incubation, 5-FU, cisplatin, and sorafenib were added for 48 h. After 48 h, cells were fixed with 4% PFA for 10 min at room temperature and washed twice with DPBS. Hoechst 33,342 was used for nuclear staining. To capture enough cells (> 1000) for analysis, five image fields were collected from each well, starting at the center of the well. All of the image analysis was performed using the HCS system and Harmony software. Cell counts were calculated and normalized to the control (0.5% DMSO).

### Cell death detection in spheroids

Spheroid cell death was detected using the cell-impermeant viability indicator ethidium homodimer-1 (EthD-1; Invitrogen). EthD-1 is a high-affinity nucleic acid stain that fluoresces weakly until binding to DNA and then emits red fluorescence (excitation/emission maxima ~ 528/617). Spheroids were incubated in 4 μM EthD-1 in HBM medium for 30 min at 37 °C, and images and the intensity of EthD-1 were obtained using the HCS system.

### Real-time PCR

Total RNA was isolated from cells using TRIzol® (Invitrogen) according to the manufacturer’s instructions. The reaction mixture contained RT buffer (Bio Basic, Amherst, NY, USA), dNTP solution (Bio Basic), RNasin® inhibitor (Promega), oligo (dT)^15^ primer (Bioneer, Daejeon, Korea), total RNA, and M-MLV reverse transcriptase (Invitrogen). The reaction mixtures were incubated at 37 °C for 1 h, and the transcription reaction was terminated by heating the mixture to 95 °C for 5 min and then rapidly cooling it on ice. PCR reactions were performed in 96-well plates in a mixture composed of cDNA, SYBR Green master mix (Applied Biosystems, Waltham, MA, USA), primers, and DEPC using a StepOnePlus real-time PCR system (Applied Biosystems). The reaction conditions were 95 °C for 10 min, followed by 40 cycles of 95 °C for 15 s and 60 °C for 1 min. The threshold cycle (CT) was defined as the fractional cycle number at which the fluorescence passes the fixed threshold. CT values were calculated according to the mathematical model *R* = 2 − ΔΔCT, where ΔCT = CT_target gene_ − CT_GAPDH_, and ΔΔCT = ΔCT_test_ − ΔCT_control_. CT values were normalized to GAPDH expression. All real-time PCR was performed in triplicates, and the data are presented as the mean ± standard deviation (SD). All primers were designed and purchased from Bioneer.

### Statistical analysis

All experiments were performed at least three times. The results are expressed as the mean ± SD. Statistical analyses were performed using the Student’s *t*-test.

## Results

### Characterization of primary cultured HCC cells as original HCC

As we aimed to develop a platform to select optimized anti-cancer drugs to provide more informative data for clinical drug application, we isolated primary HCC tumors from liver resection specimens of liver cancer patients. From these, we selected two new HCC cell lines, AMC-H1 and AMC-H2, which were obtained from HBV-infected Korean HCC patients. The characteristics of the two patients are summarized in Table 1. At the beginning of primary culture of the whole tumor after resection, hepatocytes were observed to spread out from different groups of cells. After two to three passages, the culture was almost entirely composed of hepatocytes. The hepatocyte-rich cultures were maintained for more than 50 generations in DMEM supplemented with 10% FBS.

**Table 1:** Donor characteristics at the time of HCC resection

Patient characteristics	AMC-H1	AMC-H2
Age/sex	55/Female	51/Male
HBsAg	Positive	Positive
Serum HBV DNA	1.6 × 10^6^ copies/mL	7.2 × 10^6^ copies/mL
Anti-HCV antibody	Negative	Negative
Serum α-fetoprotein	128,000 ng/mL	4.4 ng/mL
HCC Pathologic diagnosis	Multinodular confluent type	Expanding nodular type
	Vascular invasion	No vascular invasion
	Edmondson-Steiner grade 3/2^a^	Edmondson-Steiner grade 3/2^a^
	Cirrhosis	No cirrhosis
AJCC TNM staging	T3bN0M0 stage IIIB	T1N0M0 stage I

^a^Edmondson-Steiner grade worst/major

*HBV* hepatitis B virus, *HCV* hepatitis C virus, *AJCC* American Joint Committee on Cancer

**Fig. 1:**
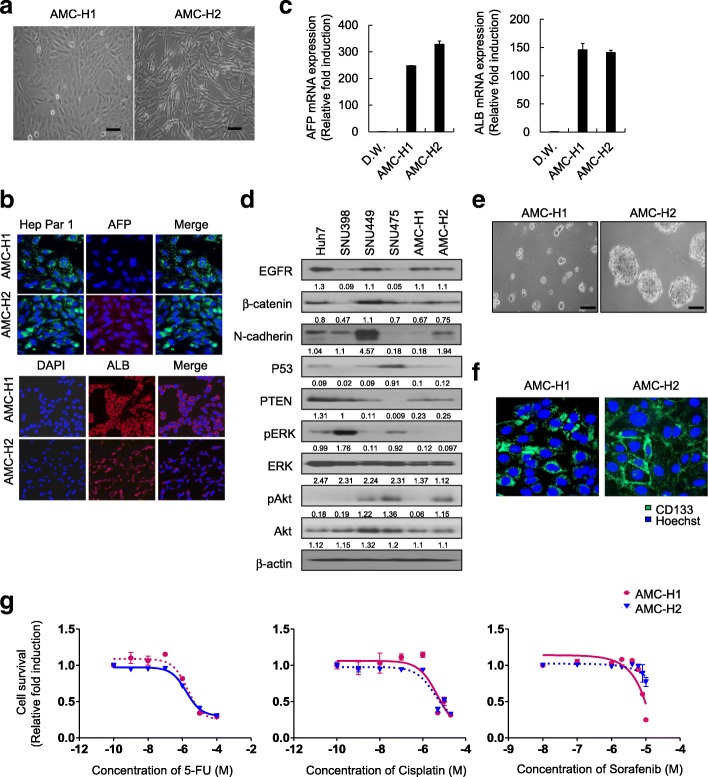
Characterization of primary HCC cells. **a** The morphology of AMC-H1 and AMC-H2 HCC cells in monolayer culture condition. **b** AFP, albumin, and HepPar-1 immunostaining of AMC-H1 and AMC-H2 cells to examine the cellular origin of primary HCC cells. **c** AFP and albumin mRNA expression levels in AMC-H1 and AMC-H2 HCC cells. **d** Expression levels of EGFR, β-catenin, N-cadherin, p53, PTEN, ERK and Akt in AMC-H1 and AMC-H2 cells were measured by western blot analysis. Activation of signaling pathways was examined by measuring phosphorylation of ERK and Akt. **e** Spheroid cultures of AMC-H1 and AMC-H2 cells in stem cell permissive medium for 5 day. The expression level of each molecule was quantitated by densitometric analysis. **f** CD133 immunostaining of AMC-H1 and AMC-H2 cells. **g** Dose response curve of AMC-H1 and AMC-H2 cells treated with 5-FU, cisplatin, and sorafenib at the indicated concentrations for 48 h under monolayer culture conditions. Bright field images were obtained using a Zeiss microscope. The fluorescence images were obtained and analyzed using the Operetta® High Content Screening (HCS) system. Data are shown as the mean ± SD of triplicate experiments. Scale bar = 200 μm

To determine whether the established cell lines were hepatocytes, we observed the morphology of AMC-H1 and AMC-H2 cells. During the proliferation phase, AMC-H1 cells appeared as a homogenous cell population with an epithelia-like or polygonal shape and clear nuclei with multiple nucleoli. The cells grew in a pavement-like arrangement with a distinct border as a monolayer. By contrast, AMC-H2 cells were spindle-shaped rather than polygonal (Fig. 1a).

To determine whether both primary HCC cell lines maintained the original HCC characteristics, we performed immunostaining with the hepatocyte-specific markers Hepatocyte Specific Antigen (Hep Par-1), alpha fetoprotein (AFP), and albumin. Hep Par-1 was expressed in both patient-derived cell lines, and AFP expression was significantly higher in AMC-H2 than in AMC-H1. Albumin was expressed in both cell lines but at higher levels in AMC-H1 (Fig. 1b). These results suggest that AMC-H1 and AMC-H2 have properties of hepatocytes but different levels of AFP and albumin expression.

To assess the liver-specific functional competence, AFP and albumin mRNA expression levels were analyzed by real-time PCR. The mRNA expression of AFP and albumin differed between AMC-H1 and AMC-H2. AFP mRNA was weakly expressed in AMC-H1 but was strongly expressed in AMC-H2. The pattern of mRNA expression in both AFP and albumin was consistent with the immunostaining results (Fig. 1c).

Both patients of established cell lines showed detectable HBV DNA in their blood at the time of resection. We assessed HBV DNA and hepatitis B surface antigen (HBsAg) in the culture supernatant to assess whether the two cell lines retained stable HBV infection. As shown in Additional file 1: Table S1, HBV DNA was still detected in both cell lines even after long periods in serial culture, although copy number (CN) was decreasing. We attempted to confirm the presence of whole genome HBV DNA in the cell lysates of the cell lines (data not shown), but our attempts were unsuccessful. Our data, nonetheless, suggest that AMC-H1 and AMC-H2 have at least part of the HBV DNA S gene region stably integrated into their genome and secrete HBV DNA continuously despite a long period of serial passaging. By contrast, we were unable to detect HBsAg in culture supernatants from either cell line (Additional file 1: Table S1). These results suggest that AMC-H1 and AMC-H2 cannot produce infectious intact virion despite the integration of at least some of the HBV DNA into the genomes.

In the wound healing assay, migration from the wound edge was greater and faster in AMC-H2 than in AMC-H1 (AMC-H1 vs AMC-H2; 100 vs 250 μm in 24 h; (Additional file 2: Figure S1)).

Collectively, these results demonstrate that the two cell lines have similar proliferation abilities, HBV DNA and HBsAg secretion, and expression of AFP and albumin but different morphology, metastatic potential, and population of liver cancer stem cells (LCSCs), suggesting that AMC-H2 is more invasive than AMC-H1.

### Profiles of tumor-related properties in AMC-H1 and AMC-H2

EGFR, β-catenin, p53, and PTEN are the most common known abnormalities in HCC [1] . Therefore, we investigated the expression levels of these factors in the new cell lines as well as several other HCC cell lines (Huh7, SNU398, SNU449, and SNU475). EGFR was expressed in both AMC-H1 and AMC-H2 at similar levels. β-catenin was not overexpressed in either of the two new cell lines compared to other HCC cell lines. The tumor suppressor p53 and PTEN were expressed at very low levels in both cell lines, potentially providing a survival benefit. We also examined N-cadherin expression, which is associated with epithelial-mesenchymal transition and metastasis, and AMC-H2 showed higher N-cadherin expression than AMC-H1. This finding was consistent with the wound healing assay, which showed higher migration potential in AMC-H2 than AMC-H1. In addition to the expression of cancer-related genes, we determined whether any signaling pathways were dominantly activated. Specifically, two major receptor-mediated activation pathways, RAS/RAF/MEK/ERK and PI3K/Akt, were examined. The two new cell lines did not show any activation of the RAS/RAF/MEK/ERK pathway, and only AMC-H2 showed PI3K/Akt pathway activation. Therefore, AMC-H1 and AMC-H2 had similar expression of oncogenes and tumor suppressors but different invasive capacity and activated signaling pathways (Fig. 1d).

Chromosomal alterations and instability may play important roles in hepatocarcinogenesis [2]. To evaluate genomic imbalances, we performed an SNP array using Affymetrix CytoScanTM 750 K, and submicroscopic chromosome abnormalities were analyzed by CN variations (CNVs) in each chromosome. CNVs involve relatively large regions of the genome that have been deleted or duplicated on certain chromosomes and result in chromosome gain or loss. As shown in karyotype analysis (Additional file 2: Figure S2), numerous genomic alterations were observed in most of the chromosomes of AMC-H1 and AMC-H2 (Additional file 3: Table S2, Additional file 4: Table S3).

In our previous study, we identified CD133 as a CSC surface marker through characterization of CSCs in primary HCC and found CD133^+^ primary HCC cells displayed greater tumor spheroid-forming ability and chemoresistance than CD133^−^ primary HCC cells [8]. For characterization of both of the new primary HCC cell lines, we estimated the spheroid-forming capacity of the cells in suspension culture, because CSCs have been enriched in non-adherent spheroids of breast, colon, and liver cancer cells. AMC-H2 cells displayed aggressive spheroid formation, however, AMC-H1 cells did not form spheroids (Fig. 1e). Next, we detected the expression of CD133 in AMC-H1 and AMC-H2 HCC cells. Immunohistochemical analysis revealed that expression of CD133 was dominantly observed at the membrane of AMC-H2 HCC cells, whereas CD133 expressed occurred only in the cytoplasm of AMC-H1 HCC cell spheroids (Fig. 1f).

### Comparison of sensitivity to anti-cancer drugs in monolayer cultured AMC-H1 and AMC-H2 cells

Due to the observed differences in activated signaling pathways, genomic alterations, and CD133 status between the two cell lines, we assessed the sensitivity of the AMC-H1 and AMC-H2 HCC cell lines to chemotherapy in the monolayer culture system. Sorafenib, 5-FU, and cisplatin, which are standard therapies for advanced HCC patients in South Korea, were incubated with both cell lines. AMC-H1 and AMC-H2 HCC cells displayed similar half maximal inhibitory concentrations (IC_50_) to 5-FU, cisplatin, and taxol and just slightly different IC_50_ value for sorafenib (Fig. 1g, Table 2).

**Table 2:** Half maximal inhibitory concentrations (IC_50_) of anti-cancer drugs in AMC-H1 and AMC-H2 primary HCC

	IC_50_(μM)		IC_50_ER
	AMC-H1	AMC-H2	
5-FU	2.05	1.71	1.198830409
Cisplatin	4.91	3.71	1.323450135
Taxol	0.125	0.127	0.984251969
Sorafenib	7.92	21.08	0.375711575

^a^*IC*_*50*_*ER:* IC_50_ with enhancement ratio (AMC-H1/AMC-H2)

### Comparison of sensitivity to anti-cancer drugs in tumor spheroids of AMC-H1 and AMC-H2 cells

**Fig. 2:**
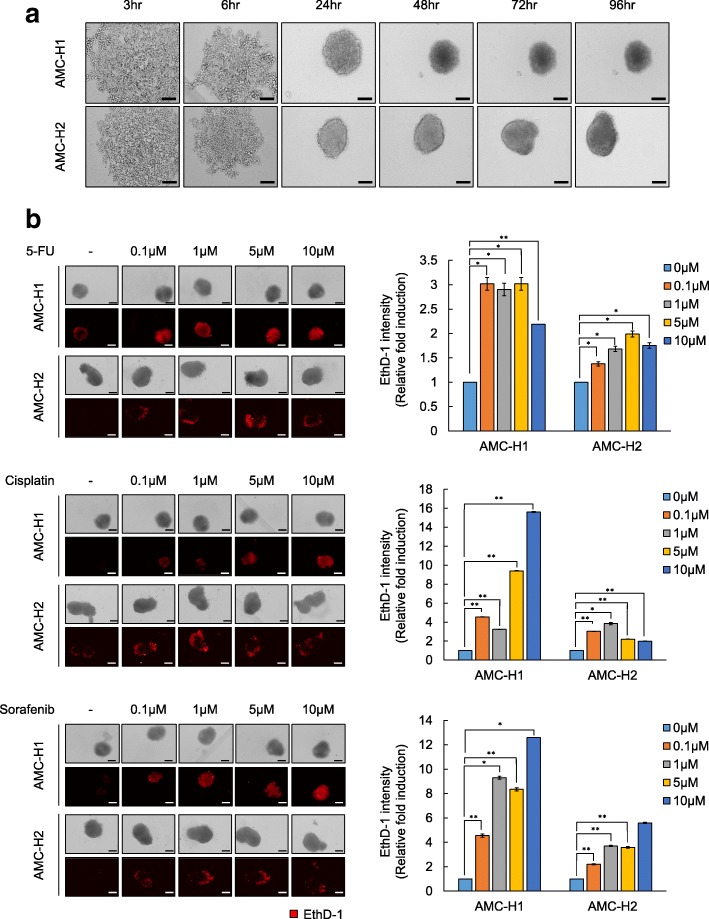
Kinetics and drug sensitivity of tumor spheroids from primary HCC cells. **a** Kinetics of tumor spheroids from AMC-H1 and AMC-H2 cells at the indicated times. Cells were cultured in 96-well ultra-low attachment (ULA) plates for 96 h. **b** Drug sensitivities in tumor spheroids of AMC-H1 and AMC-H2 HCC cells. Cells were cultured in 96-well ULA plates for 3 day and then treated with 5-FU, cisplatin, and sorafenib at the indicated concentrations for 7 day. Then, tumor spheroids were stained with ethidium homodimer-1 (EthD-1; left panel), and the staining intensity was analyzed (right panel). All images were obtained and analyzed using the HCS system. Data are shown as the mean ± SD of triplicate experiments. Scale bar = 200 μm. *p < 0.05, **p < 0.005

Since 3D tumor spheroids can reflect in vivo features of tumors better than conventional monolayer cultured cells, we posit that tumor spheroids will significantly contribute to the development of optimized treatment for patients with hepatocellular carcinoma medicine. Kinetics of the rate of spheroid assembly showed that AMC-H2 cells achieved strong cohesion in short periods of time, whereas AMC-H1 cells displayed limited ability to enhance spheroid stiffness (Fig. 2a). For drug efficacy tests on the spheroids, generation of homogenous sized, single spheroids of patient’s cells in multi-well plates will be essential for comparison of the responses under various conditions of compounds and concentrations. To determine the feasibility of such constraints, we successfully generated homogenous sized, single spheroids in multi-well plates. Bright-field images were obtained by Operetta HTS system for just 10 min. Hence, we established a simple method for highly reproducible (< 10% size variation) spheroids and associated high-speed image acquisition. To detect cell death in the spheroids, we used EthD-1, which incorporates into the DNA of dead cells and emits fluorescence. We then analyzed the intensity of EthD-1 staining to quantify the concentration of cell damage (Fig. 2b).

Interestingly, the tumor spheroids of AMC-H1 cells and AMC-H2 cells displayed different drug sensitivities in monolayer cultures (Fig. 2b). Spheroids with AMC-H2 cells showed strong resistance to sorafenib, 5-FU, and cisplatin, whereas these drugs significantly reduced cell survival in spheroids of AMC-H1 cells. Differences in drug reactivity against 5-FU and cisplatin between AMC-H1 cells and AMC-H2 cells, which were not apparent under monolayer culture conditions, were observed under 3D culture conditions. In general, drugs must diffuse only a short distance across the cell membrane to reach the target when tested in monolayer culture condition, whereas the 3D culture conditions are more realistic, and the drugs must diffuse across multi layers of cells to reach its target. Thus, it is generally thought that chemoresistance will be greater in 3D cultures than in monolayer cultures. Unexpectedly, however, drug reactivity was more sensitive in 3D culture conditions than in monolayer culture conditions for the HCC cell lines. These results demonstrated that sorafenib has the most efficient anticancer effect on AMC-H1 cells and that none of the drugs tested were an effective medicine for AMC-H2 cells.

### Formation of MCTS and their sensitivity to anti-cancer drugs in primary HCC cells

The 3D tumor spheroid represents the tumor microenvironment, with respect to gradient distribution of oxygen, metabolites, and nutrients as well as drug penetration, similar to tissues in vivo [9]; however, these structures lack other key component of tumor tissues. Specifically the tumor microenvironment includes epithelial cells, fibroblasts, activated hepatic stellate cells, infiltrating immune cells, the endothelial vascular system, and ECM molecules, such as collagen and integrin [10]. To bridge this gap, we previously established simple MCTS models using HCC cell lines and either LX2 (human hepatic stellate cells, HSCs), WI38 (human fibroblasts), or HUVEC (vascular endothelial cells) stromal cells at a 1:1 ratio to mimic tumor heterogeneity observed in vivo [11]. This system was limited as only one type of stromal cells were mixed with the HCC cells.

**Fig. 3:**
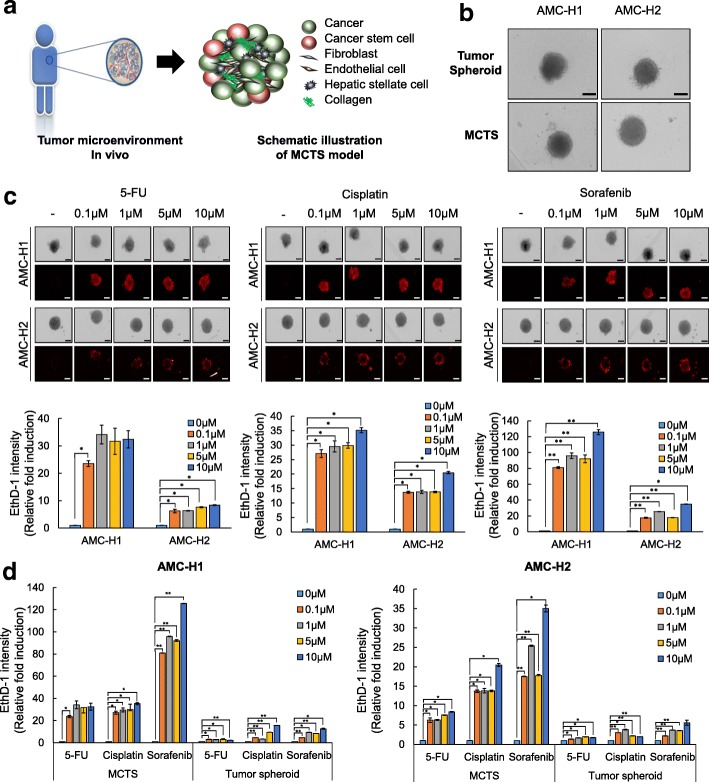
MCTS formation and drug sensitivity of primary HCC cells. **a** Schematic of MCTS model representing the in vivo microenvironment. **b** Morphology of tumor spheroids and MCTS of AMC-H1 and AMC-H2 HCC cells. **c** Drug sensitivities in MCTS of AMC-H1 and AMC-H2 HCC cells grown with hepatic stellate cells (HSC; LX2), fibroblasts (WI38), and endothelial cells (HUVECs). Cells were cultured in 96-well ULA plates for 3 day and then treated with 5-FU, cisplatin, and sorafenib at the indicated concentrations for 7 day. Then, MCTS were stained with EthD-1 (upper panel), and the staining intensity was analyzed (lower panel). **d** EthD-1 intensity of tumor spheroids and MCTS composed with AMC-H1 or AMC-H2 after treatment with the indicated drugs. All images were obtained and analyzed using the HCS system. Data are shown as the mean ± SD of triplicate experiments. Scale bar = 200 μm. *p < 0.05, **p < 0.005

In this study, we sought to generate patient-derived MCTS as a model for clinical application for investigating drug sensitivity (Fig. 3a). To this end, AMC-H1 cells and AMC-H2 cells were co-cultured with three types of stromal cells (LX2, WI38, and HUVEC) under 3D culture conditions. AMC-H1 and AMC-H2 MCTS exhibited a more compact and sharper morphology than tumor spheroids (Fig. 3b). In addition, the intensity of EthD-1 staining was analyzed after treatment with 5-FU, cisplatin, and sorafenib at the indicated concentrations. Interestingly, we obtained similar results for the responsiveness to the three chemotherapy agents for both MCTS and tumor spheroids. Specifically, sorafenib had the most efficient anticancer effect on AMC-H1 cells, and none of the reagents tested were effective for AMC-H2 cells (Fig. 3c).

With respect to drug sensitivity, the AMC-H1 MCTS and AMC-H2 MCTS were more sensitive relative to simple tumor spheroids. Particularly, susceptibility to sorafenib, which was not observed in AMC-H2 spheroids, was observed in AMC-H2 MCTS, confirming that sorafenib is the best chemotherapy for the AMC-H2 cells (Fig. 3d).

These results showed that sensitivity to chemotherapies varies depending on the culture methods, despite the drug sensitivity observed for the primary HCC cells. Furthermore, these findings suggest that a 3D architecture tumor microenvironment and crosstalk between tumor and stromal cells affect sensitivity to chemotherapies.

### Comparison of drug sensitivity of monolayer cultured cells, tumor spheroids, and MCTS of primary HCC cells

AMC-H1 and AMC-H2 HCC cells were obtained from liver patient samples and were then stabilized as cancer cell lines. Therefore, we investigated the drug sensitivities of monolayer cultured cells, tumor spheroids, and MCTS of eight primary HCC cells that were derived from liver patients and passaged only 3-6 times. When primary HCC cells were cultured, multiple cell types grew; however, only HCC cells survived in culture without stromal cells after 3-6 passages under hepatocyte-specific culture conditions. Prior to investigating the drug sensitivities of these cell conditions, we examined the ability of these cells to form tumor spheroids and MCTS (Additional file 2: Figure S3A). Six of eight tested primary HCC cells formed tightly compacted spheroids; however, 8513B and 51532B formed spheroids with loose aggregate morphology. In the presence of stromal cells, MCTSs of all primary HCC cells displayed tight and sharp morphology compare to tumor spheroids, perhaps due to the ability of stromal cells to support cell-cell interactions and facilitate HCC assembly. Furthermore, although expression of liver or liver cancer markers differs between primary HCC cells, eight cultures of primary HCC cells exhibited albumin and HepPar-1 expression in the cytosol (Additional file 2: Figure S3B) as well as AFP and albumin mRNA expression (Additional file 2: Figure S3C).

With our established monolayer cultured cell system, tumor spheroids, and MCTS, drug sensitivities were investigated and compared between various primary HCC cells (Table 3). The analysis methods differed between the 2D and 3D culture systems. In the monolayer culture system, nuclei were stained, cell numbers were determined, and survival rates were given relative to control (0.1% DMSO). On the other hand, tumor spheroids and MCTS were stained with EthD-1 for detecting cell death, and cell death rates were reported relative to control (0.1% DMSO). Due to these differences, the sensitivities to anti-cancer drugs were not compared with specific inhibitory concentrations, such as IC_50_. Instead, drug sensitivities were assigned categories as follows: “-” means >90% cell survival in monolayer cultures and <10% cell death in tumor spheroids and MCTS, “+” means > 70% but < 90% cell survival in monolayer cultures and < 30% but > 10% cell death in tumor spheroids and MCTS, and “++” means < 50% but > 30% cell survival in monolayer cultures and < 50% but > 30% cell death in tumor spheroids and MCTS.

**Table 3:** Drug sensitivities in monolayer culture system, tumor spheroids, and multicellular tumor spheroids (MCTSs) of patient-derived cells

Cells	Spheroid formation	Drug sensitivity								
		Monolayer culture			Tumor spheroids			MCTS		
		5-FU	Cis	Sor	5-FU	Cis	Sor	5-FU	Cis	Sor
103209	Compacted spheroid	++	–	–	–	–	–	+	–	+
101509	Compacted spheroid	+	+	–	+	–	–	+	–	–
116261	Compacted spheroid	++	+	–	–	–	–	–	–	–
8513B	Loosely compacted aggregates	+	–	–	++	+	–	–	–	–
51532B	Loosely compacted aggregates	+	+	–	++	+	+	+	–	++
21612B	Compacted spheroid	+	–	–	–	–	–	–	–	–
35372B	Compacted spheroid	+	–	–	++	–	+	+	+	–
27273B	Compacted spheroid	++	–	–	+	+	–	+	–	–

Drug sensitivity of 2D were compared to each other, and drug sensitivity of 3D and MCTS were compared together to each other. -; no drug sensitivity, +; middle of drug sensitivity, ++; high of drug sensitivity

^a^*Cis* Cisplatin, *Sor* Sorafenib

As shown in Table 3, all primary HCCs responded sensitively to 5-FU in monolayer culture, while only half of the primary HCCs responded weakly to 5-FU in tumor spheroids and MCTS system. The selective response to 5-FU was most pronounced in MCTS culture system. Response to cisplatin showed a totally different pattern between the 2D and 3D culture systems, and not all of the primary HCCs MCTS exhibited sensitivity to cisplatin. Thus, all of the primary HCCs MCTS displayed the strongest cisplatin resistance among the three culture systems. By contrast, primary HCCs MCTS displayed the most selective response to sorafenib. None of the primary HCCs in monolayer culture system responded to sorafenib treatment, while the primary HCCs tumor spheroids responded only weakly to sorafenib.

From these data, we conclude that each primary HCC cell culture showed different drug sensitivities depending on the culture methods. Clearly, simplifying the assay system to monolayer cultures will not be beneficial for optimized treatment for patients with hepatocellular carcinoma therapy of HCC as the resulting data cannot be utilized for translational research. Collectively, we suggested MCTS culture systems are the best methodology to screen for optimized therapy for patients with hepatocellular carcinoma, because tumor spheroids strikingly mirror the 3D cellular context and therapeutically relevant pathophysiological gradients and heterogeneity of in vivo tumors.

## Discussion

HCC is a highly heterogeneous disease in terms of its molecular profiles, and such heterogeneity has been a major obstacle to the study of tumorigenesis and identification of therapeutic targets. Therefore, the establishment of cell lines derived from HCC patients, especially from HBV-related HCC is required for the in vitro study of HCC, as HBV is the most common etiology of HCC worldwide. In this study, we established two new HBV-associated, patient-derived HCC cell lines (AMC-H1 and AMC-H2) and characterized the morphology; HBV DNA secretion; cellular properties, such as invasiveness; cytogenetic; and molecular features of these cell lines (Fig. 1).

AMC-H1 and AMC-H2 both expressed AFP and more importantly secreted HBV DNA stably. AFP expression levels were higher in AMC-H2 than in AMC-H1; yet, the AMC-H1 donor showed much higher serum AFP levels at the time of tumor resection than the AMC-H2 donor. We cannot explain this discrepancy, but we assume that both cell lines were altered with respect to their capacity to express AFP during the establishment of the cell lines (Fig. 1b-c). In terms of HBV DNA, we were unable to detect the whole genome of HBV in cell lysates; however, stable HBV DNA expression in these cells lines must occur due to HBV DNA incorporation, at least in part, into the chromosomes of both cell lines (Additional file 1: Table S1, Additional file 3: Table S2, Additional file 4: Table S3). Furthermore, we did not detect HBsAg in either AMC-H1 or AMC-H2 supernatants. One explanation for this could be that incorporation of only partial HBV DNA into the host genome precluded expression of this antigen. Another explanation is that HBsAg escape mutations [12–14] occurred during the in vitro establishment period, although this possibility is quite low. Collectively, both AMC-H1 and AMC-H2 expressed AFP and HBV DNA stably after a long period of serial culture, but infectious intact HBV virion was not produced. Further virological study is needed to assess the mechanism of stable HBV DNA secretion from our cell lines and its usefulness in the study of HBV-related HCC studies.

We also examined the tumor-related genes and signaling pathways in these cell lines. EGFR [15] and β-catenin [16] are well-known factors involved in HCC tumorigenesis, but neither was overexpressed in the cell lines. In HCC, it has been reported that EGFR mutation is quite rare, but β-catenin mutation is more frequent. Thus, specific examination of β-catenin mutation in AMC-H1 and AMC-H2 will be required in future studies (Fig. 1d). The representative tumor suppressors, p53 and PTEN, were also examined in our new cell lines, and both tumor suppressors were expressed at a low level compared to expression in other HCC cell lines. Among receptor-mediated signaling pathways, the Ras/Raf/MEK/ERK pathway was not activated in either cell line, but the PI3K/Akt pathway was activated in the AMC-H2 cell line. These results suggest that other signaling pathways such as Wnt/β-catenin [17, 18], VEGF [19], FGF, or IGF [3] may play a more important role in the proliferation of our cell lines, and as such, further study is warranted.

Chromosome instability is a hallmark of solid tumors and is a characteristic genetic alteration of HCC [4]. It has been reported that HBV-related HCC have common chromosomal abnormalities, including gains of 1q, 6p, 8q and 17q and losses of 1p, 4q, 6q, 8p, 9p, 13q, 16q, and 17p [5, 6]. As shown in Tables 2, 3, these common aberrations were also found in AMC-H1 and AMC-H2 and chromosomal aberrations observed in AMC-H1 and AMC-H2 were markedly divergent even at the same chromosome loci. In addition to common chromosomal abnormalities, analysis of an SNP array showed that AMC-H1 and AMC-H2 have numerous genomic alterations on most chromosomes. Furthermore, chromosomal alterations varied between the two cell lines; for instance, AMC-H2 mainly had chromosome losses while AMC-H1 did not. In general, chromosome gains frequently occur by duplication of genetic regions; however, chromosome gains by duplication have little oncogenic potential, as oncogenic drivers are typically the result of chromosome gains by focal amplification (CN state = 6-7). AMC-H1 and AMC-H2 have highly duplicated genetic loci but no focal amplification. Chromosome gains in AMC-H1 were mainly related to proliferation, while AMC-H2 showed amplification in regions containing survival genes. Chromosome amplification may play important roles in the survival of both cell lines by different signaling pathways. LOH is the common chromosomal alteration in HCC, and both cell lines have LOH on most of their chromosomes. Common LOHs observed in AMC-H1 and AMC-H2 are IGF2R and p53, which have clinical significance in HCC, as both are known to predict poor clinical outcomes in surgically resected primary HCC [7, 20]. In addition to these common LOH, AMC-H1 and AMC-H2 have unique and divergent LOH, which may confer distinct properties to each of these cell lines.

Because of the molecular heterogeneity of HCC, proper in vitro systems are required to study HCC pathogenesis [21]. Many research groups reported the establishment of HCC cell lines from HBV-infected patients. Although some of these groups observed HBV DNA integration into the host genome [22–26], only a few HCC lines secrete HBV DNA or HBsAg stably [27]. In this study, we established two new HCC cell lines from HBV-infected Korean HCC patients and characterized their properties in detail. The cell lines, designated as AMC-H1 and AMC-H2, were very different from one another in morphology, signaling pathways, and chromosomal aberrations but expressed AFP and albumin and more importantly secreted HBV DNA continuously in their supernatant.

In recent years, a paradigm shift from 2D to 3D cell culture techniques has occurred because culturing cells in 2D result in unnatural cell attachment, whereas culturing cells in 3D results in formation of natural cell-to-cell attachments. Accordingly, 3D cell cultures have been shown to have dramatic effects on cell polarity and differentiation as well as signaling cascades and gene expression profiles compared with that in monolayer culture. Moreover, cells grown in 3D are more valid for discovering new drugs to treat cancer, in part, because cells grown in 3D cultures form multilayers of cells, whereas cells in 2D form a monolayer of cells spread thin on a plastic surface. When testing a drug in 2D, the drug needs only to diffuse a short distance across the cell membrane to reach its target. In 3D, however, the situation is more realistic, and a drug needs to diffuse across multi layers of cells to reach its target.

Particularly, we examined MCTS models, which were necessary in order to establish an in vitro model that includes additional cell types to resemble the heterogeneity and complexity of in vivo tissue. We sought to develop this model to provide a physically relevant source for drug discovery and personalized medicine [11, 28, 29] Therefore, we compared the drug sensitivities of sorafenib, cisplatin and 5-FU in monolayer cultures, tumor spheroids, and MCTSs from AMC-H1 and AMC-H2 cell lines and primary HCC cells from liver cancer patients. The size of spheroids were diverse depend on the cells, but average volume of spheroids were 300-400 μm. Although each primary HCC cell culture showed different drug sensitivities depending on the culture method, we concluded that simplifying the conditions for a monolayer-based chemosensitivity system is not be beneficial in terms of optimized treatment for patients with hepatocellular carcinoma therapy of HCC. The key of optimized therapy of HCC stems from the difference in results of chemosensitivity between homogeneous HCC spheroids and heterogeneous MCTS-HCC models. Because we found that MCTS-HCC models exhibited clear selective response to sorafenib, cisplatin, and 5-FU as opposed to the homogeneous HCC spheroids (Fig. 3d and Table 3), we suggest that MCTS-HCC models are the best methodology to screen for optimized therapy for patient with HCC. We, however, still cannot confidently assert that MCTS-HCC models represent identical in vivo tumor microenvironments for personalized medicine of HCC, because we do not yet have consistent evidence for clinical treatment. Therefore, we are currently securing abundant patient samples in conjunction with their history of clinical treatment with patient consent in order to inform future investigations.

## Conclusions

In this study, we developed AMC-H1 and AMC-H2 HCC cell lines that may be useful models for research in HBV-related HCC pathogenesis, for identification of novel drug targets, and for cancer drug development. Furthermore, we propose that chemosensitivity assays of potential cancer drugs on patient-derived MCTSs in vitro can be used to further the personalized oncology field.

## Additional files

Additional file 1: **Table S1.** Quantification of HBV DNA and HBsAg in AMC-H1 and AMC-H2. (DOCX 25 kb)

Additional file 2: **Figure S1.** Wound healing assay of primary HCC cells. Wound healing assay. (A) A wound was introduced after cells reached 80-90% confluence. Cell migration was monitored under microscopy for the indicated time. Scale bar = 500 μm. (B) Cell growth was monitored for 7 days, and was quantitated of migration rate which is represented by % of control. **Figure S2.** Karyoviews of AMC-H1 and AMC-H2. The blue bar indicates gain, the red bar indicates loss, and the purple bar indicates LOH. **Figure S3.** Characterization of various primary HCC cells. (A) Capacity of various primary HCC to form tumor spheroids and MCTS. (B) Albumin and HepPar-1 immunostaining to examine the cellular origins of primary HCC cells. (C) AFP and albumin mRNA expression levels in primary HCC cells. (PDF 790 kb)

Additional file 3: **Table S2.** Chromosome losses detected by SNPs array in AMC-H1 and AMC-H2. (DOCX 26 kb)

Additional file 4: **Table S3.** Chromosome gains detected by SNPs array in AMC-H1 and AMC-H2. (DOCX 26 kb)

## Abbreviations

2D: Two dimensional
3D: Three dimensional
5-FU: 5-fluorouracil
AFP: Alpha-fetoprotein
ECM: Extracellular matrix
EGFR: Epidermal growth factor receptor
EthD-1: Ethdium homodimer-1
FBS: Fetal bovine serum
FBS: Fetal bovine serum
FGF: Fibroblast growth factor
HBsAg: HBV DNA and hepatitis B surface antigen
HBV: Hepatitis B virus
HCC: Hepatocellular carcinoma
HIF1: Hypoxia- inducible factor 1
HSCs: Human hepatic stellate cells
HTS: High throughput screening
HUVECs: Human umbilical vein endothelial cells
IGF: Insulin-like growth factor
LCSCs: Liver cancer stem cells
MCTSs: Multicellular tumor spheroids
PI3K: Phosphatidylinositol-4,5-bisphosphate 3-kinase
PTEN: Phosphatase and tensin homolog
SNP array: Single nucleotide polymorphism arrays
TME: Tumor microenvironments
VEGF: Vascular endothelial growth factor
