# Supplementary material for: Patient-derived multicellular tumor spheroids towards optimized treatment for patients with hepatocellular carcinoma
Source: J Exp Clin Cancer Res. 2018 May 25;37:109. doi: 10.1186/s13046-018-0752-0 (PMC5970513; doi:10.1186/s13046-018-0752-0)
Supplement: Supplementary file 1 — Table S1. Quantification of HBV DNA and HBsAg in AMC-H1 and AMC-H2. (DOCX 25 kb) [file 13046_2018_752_MOESM1_ESM.docx]

**Table S1. Quantification of HBV DNA and HBsAg in AMC-H1 and AMC-H2**

|  | **AMC-H1** | | **AMC-H2** | |
| --- | --- | --- | --- | --- |
| Test date | December. 1  2008 | June. 13  2012 | December. 1  2008 | June. 13  2012 |
| HBV DNA (copies/mL) | 8,670 | 1,550 | 2,685 | 700 |
| HBsAg (IU/mL) | NA | Negative | NA | Negative |

HBV, hepatitis B virus; HBsAg, hepatitis B virus surface antigen; NA, not applicable
