# Supplementary figures and images for: Patient-derived multicellular tumor spheroids towards optimized treatment for patients with hepatocellular carcinoma
Source: J Exp Clin Cancer Res. 2018 May 25;37:109. doi: 10.1186/s13046-018-0752-0 (PMC5970513; doi:10.1186/s13046-018-0752-0)

Figure S1

A.

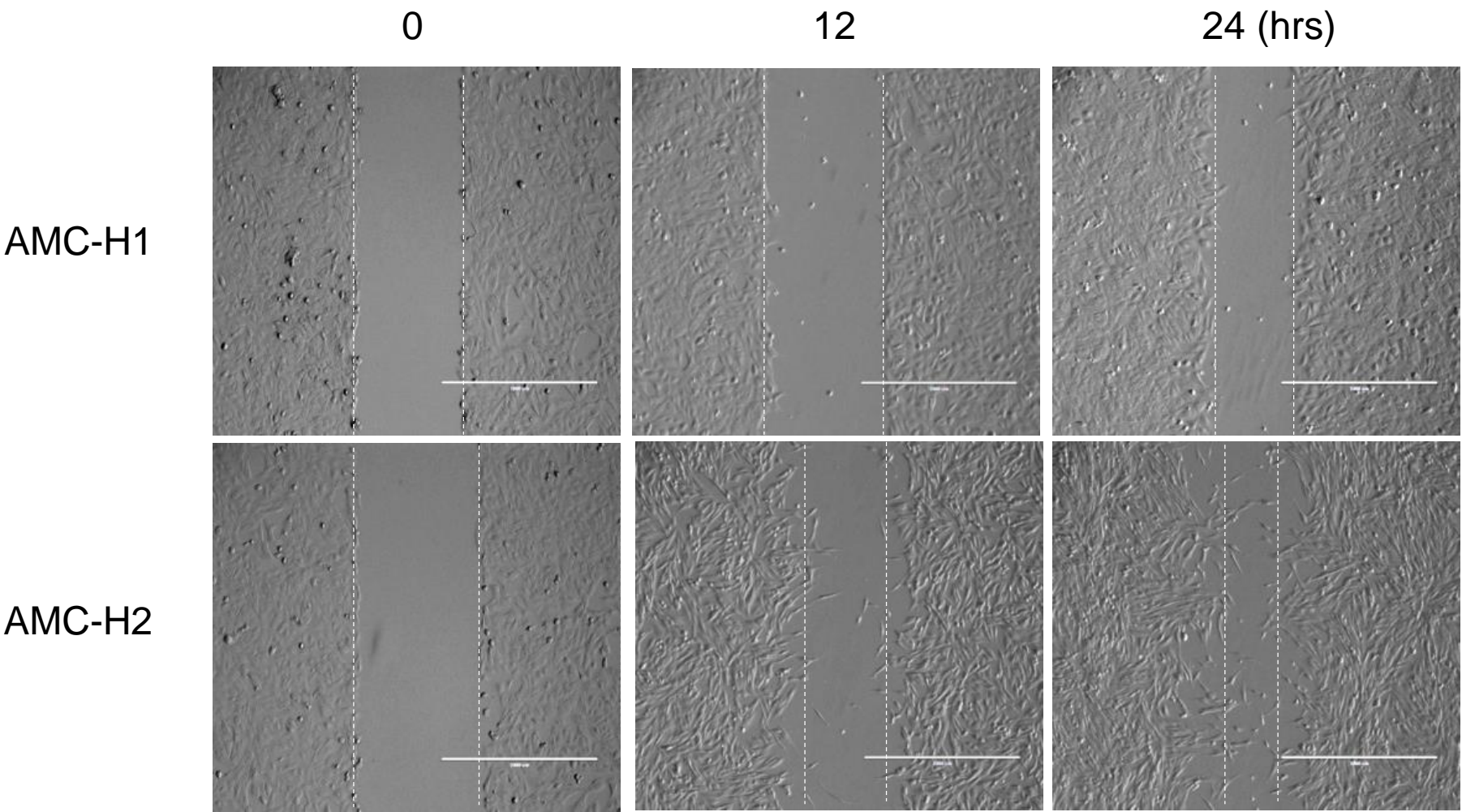

B.

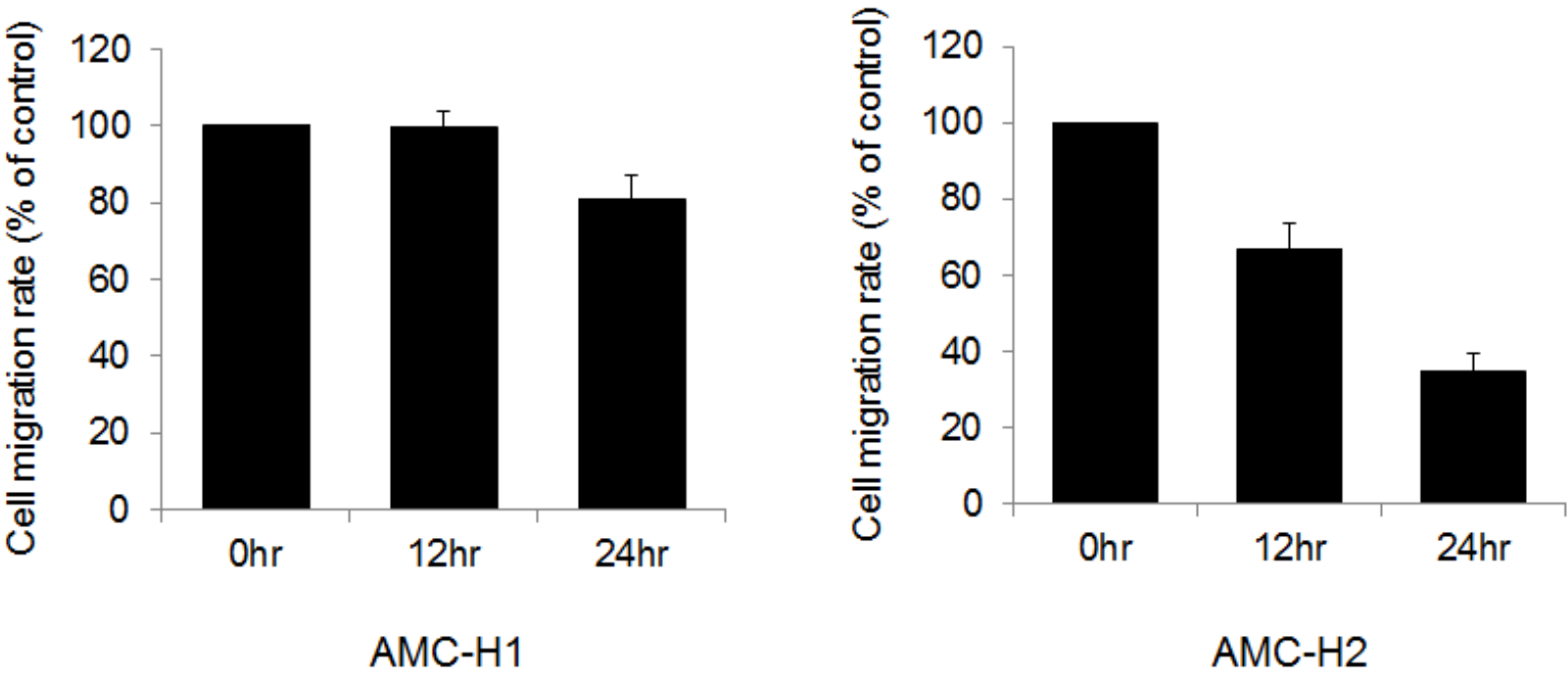

# Figure S2

AMC-H1

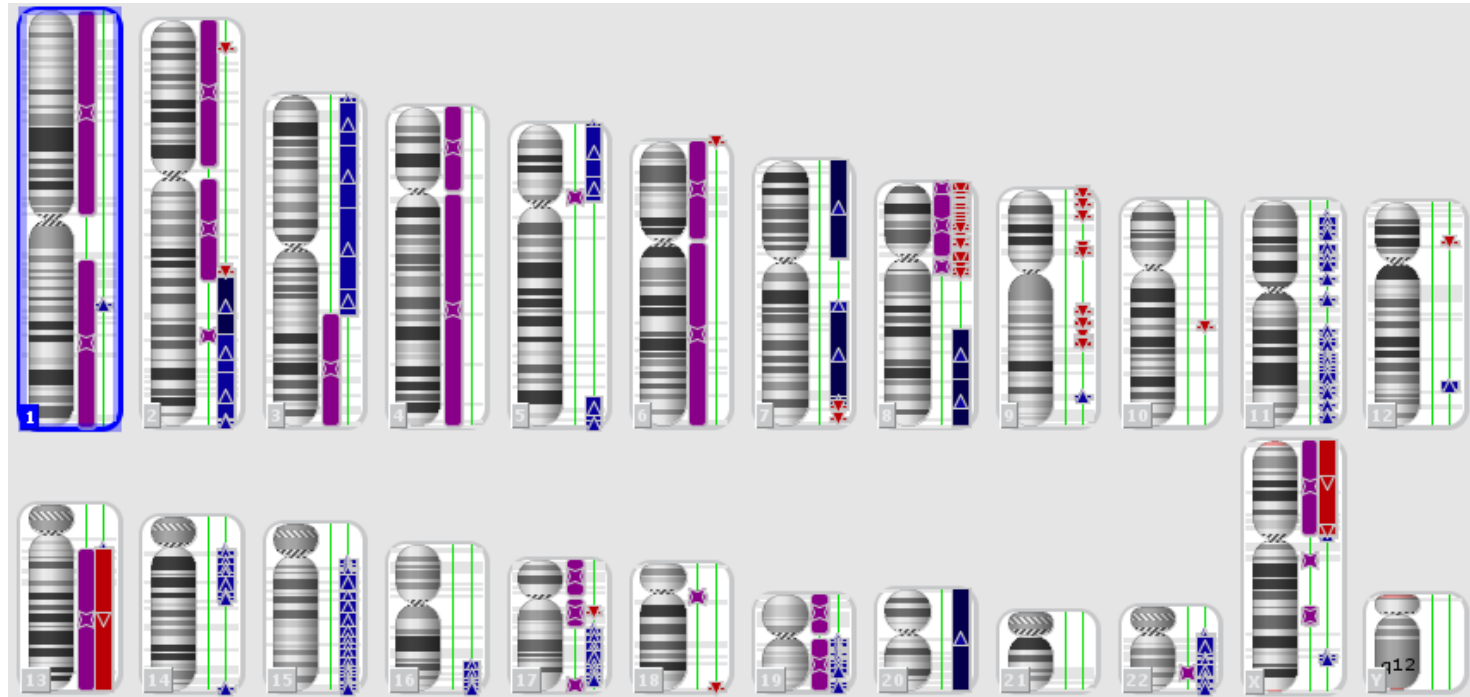

AMC-H2

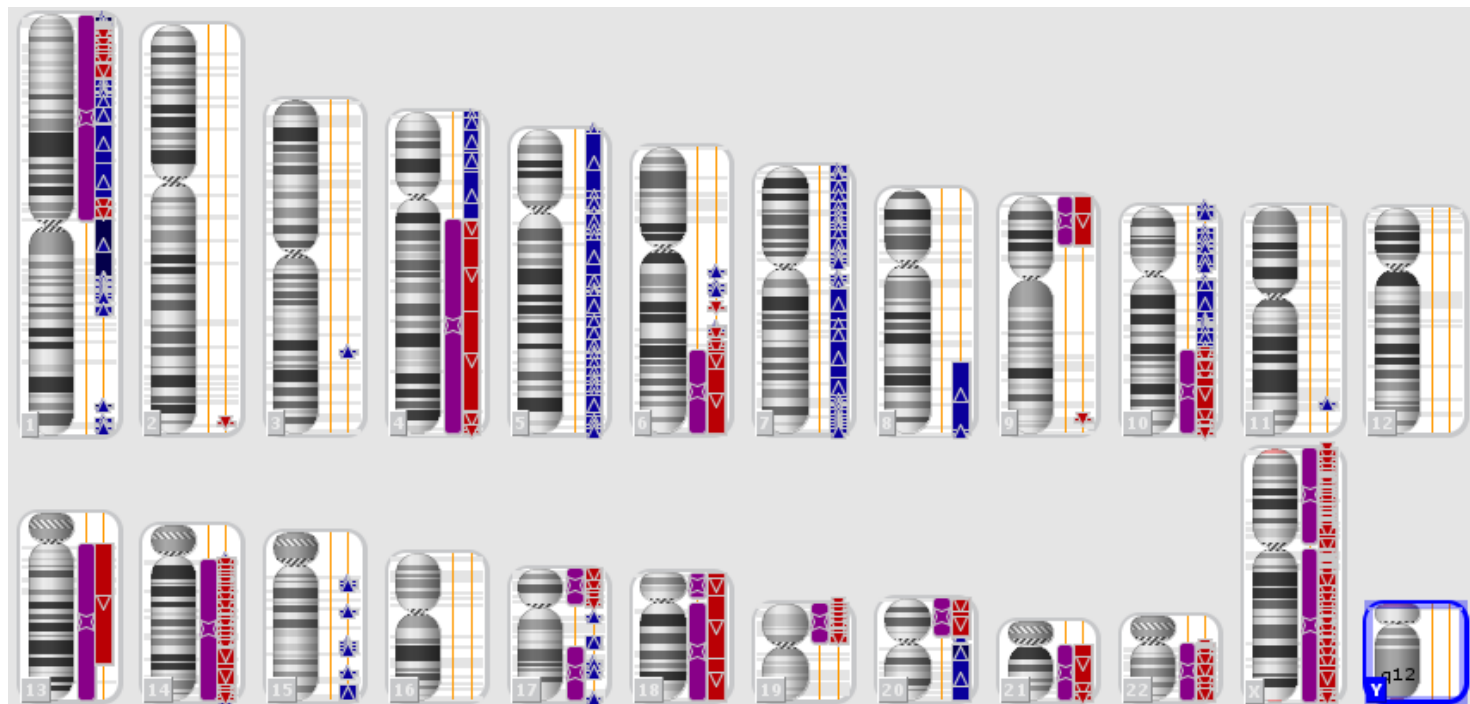

# Figure S3

A.

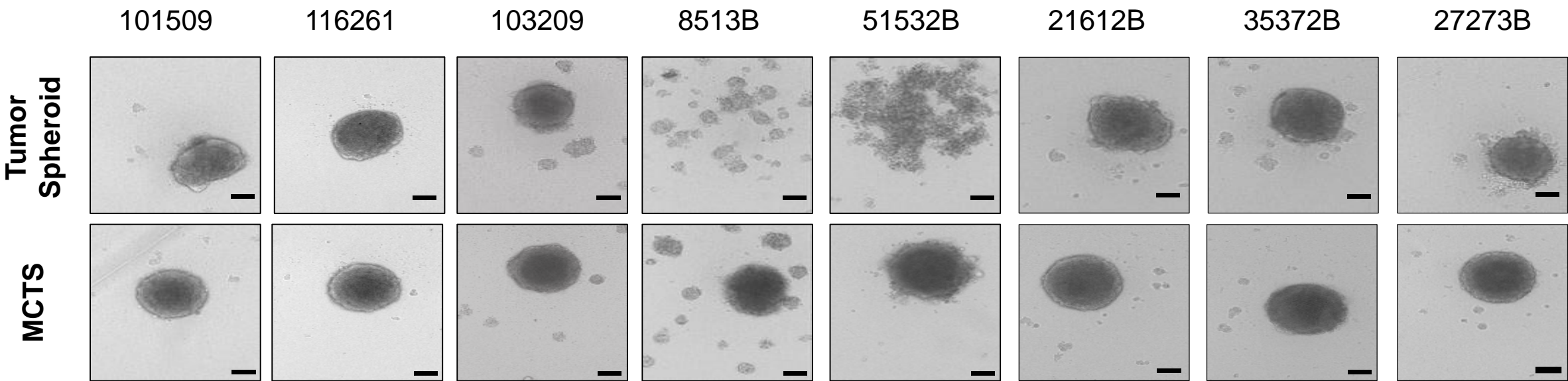

B.

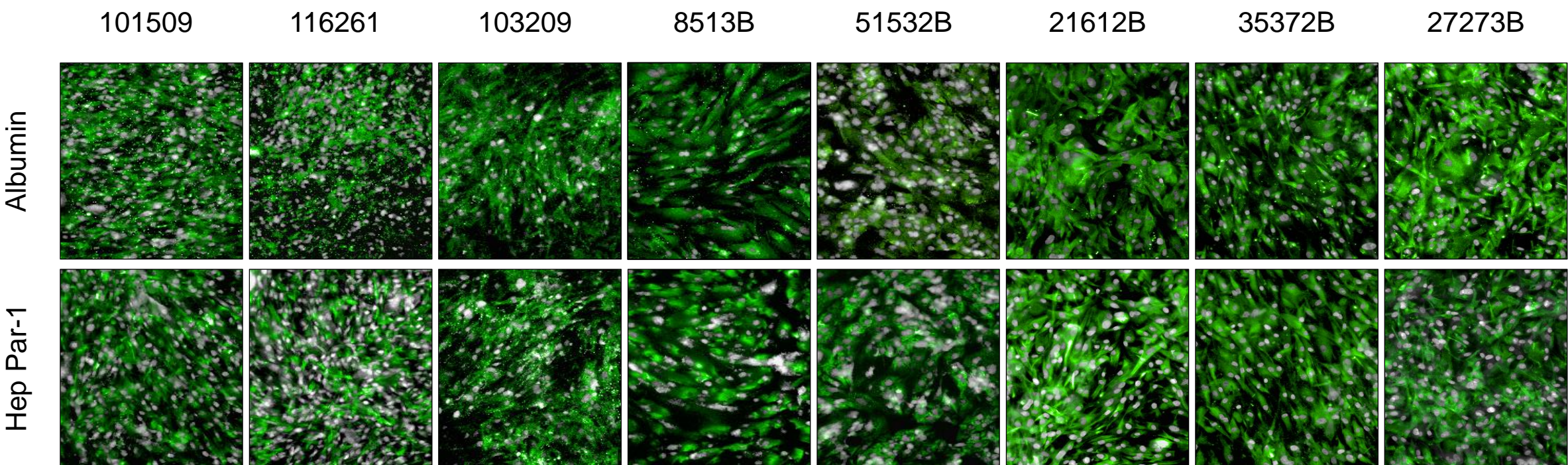

C.

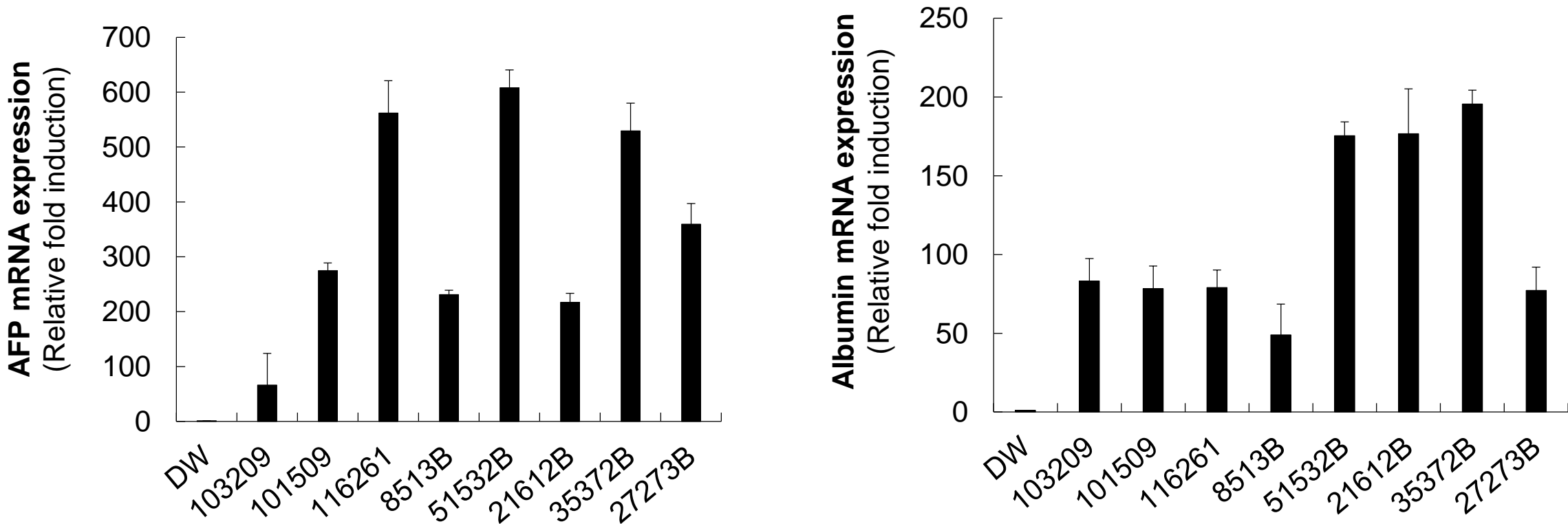

Supplement: Supplementary file 2 — Figure S1. Wound healing assay of primary HCC cells. Wound healing assay. (A) A wound was introduced after cells reached 80-90% confluence. Cell migration was monitored under microscopy for the indicated time. Scale bar = 500 μm. (B) Cell growth was monitored for 7 days, and was quantitated of migration rate which is represented by % of control. Figure S2. Karyoviews of AMC-H1 and AMC-H2. The blue bar indicates gain, the red bar indicates loss, and the purple bar indicates LOH. Figure S3. Characterization of various primary HCC cells. (A) Capacity of various primary HCC to form tumor spheroids and MCTS. (B) Albumin and HepPar-1 immunostaining to examine the cellular origins of primary HCC cells. (C) AFP and albumin mRNA expression levels in primary HCC cells. (PDF 790 kb) [file 13046_2018_752_MOESM2_ESM.pdf]
