# Supplementary material for: Patient-derived multicellular tumor spheroids towards optimized treatment for patients with hepatocellular carcinoma
Source: J Exp Clin Cancer Res. 2018 May 25;37:109. doi: 10.1186/s13046-018-0752-0 (PMC5970513; doi:10.1186/s13046-018-0752-0)
Supplement: Supplementary file 3 — Table S2. Chromosome losses detected by SNPs array in AMC-H1 and AMC-H2. (DOCX 26 kb) [file 13046_2018_752_MOESM3_ESM.docx]

**Table S2. Chromosome losses detected by SNPs array in AMC-H1 and AMC-H2**

| Chromosome No. | AMC-H1 | AMC-H2 |
| --- | --- | --- |
| 1 |  | p13-p12, p36-p35, p34 |
| 2 | q23.3, p24.3, q23.1 | q37.2 |
| 4 |  | q13.1-q34.3, q34.3-q35.2 |
| 6 | p25.3-p11.1 | q16.1, q21, q22.31-q23.2, q22.1-q22.2, q23.2-q27 |
| 7 | q35, q36.2 |  |
| 8 | p11.21-p11.23, p12, p21.3-p21.2, p23.3-p22 |  |
| 9 | p13.2, p13.3, p24.3-p24.1, q21.11-q21.2, q21.33, q22.1-q22.3 | q34.11 |
| 10 | q22.2 | p24.3-p21.1, q34.11, |
| 11 |  | q23.1-q26.13, q26.2-q26.3 |
| 12 | p12.1 |  |
| 13 | q12.1-q34 | q11-q31.3 |
| 14 |  | q11.2-q12, q13.1-q21.1, q21.3-q22.1, q22.3-q23.1, q23.2-q32.2, q32.33 |
| 17 | q12.1-q34 | p11.2, p13.3-p11.2 |
| 18 | q23 | p11.32-p21.2, q21.2-q23 |
| 19 |  | p12-p11, p13.3, p13.2-p12 |
| 20 |  | p13-p11.21 |
| 21 |  | q11.2-q22.3 |
| 22 |  | q11.1-q11.21, q11.21-q13.33 |
