# Supplementary material for: Patient-derived multicellular tumor spheroids towards optimized treatment for patients with hepatocellular carcinoma
Source: J Exp Clin Cancer Res. 2018 May 25;37:109. doi: 10.1186/s13046-018-0752-0 (PMC5970513; doi:10.1186/s13046-018-0752-0)
Supplement: Supplementary file 4 — Table S3. Chromosome gains detected by SNPs array in AMC-H1 and AMC-H2. (DOCX 26 kb) [file 13046_2018_752_MOESM4_ESM.docx]

**Table S3.** **Chromosome gains detected by SNPs array in AMC-H1 and AMC-H2**

| **Chromosome No.** | **AMC-H1** | **AMC-H2** |
| --- | --- | --- |
| 1 | q25.1, q34-q37.3 | p33-p12, p34.3-p34.1, p36.32-p36.13, p36.33-p36.32, q23.2, q23.3-q24.1, q24.2, q42.2, q43-q44 |
| 2 | q37.3, q32.1-q34, q23.1-q32. |  |
| 3 | p14.1-q22.1 | q24-q25.1 |
| 4 |  | p16.3-p15.1, p14-q13.1 |
| 5 | p15.33-p12, q34-q35.3 | q33.1-q31.2, p15.33-p13.2, q35.3, q12.3- q14.3-q21.1, p13.2-p13.1, q12.1, q11.1-q12.1, q33.1-q33.3, q23.2-q31.3, p13.1-p12, q31.1, q35.1-q35.2, q21.1-q22.2, q23.1-q23.2, q31.2-q31.3, q31.3-q32, q22.2-q23.1, q33.3-q35.1, q15 |
| 6 |  | q22.31-q21, q14.1, q13, q14.3 |
| 7 | q21.2-q34, q21.11-q21.13, p22.3-p11.2, q34-q35 | p11.2, p13-p12.2, q36.3, p22.1, p21.3-p21.2, p14.1-p13, q11.23-q21.2, p14.2-p14.1, p14.3-p14.2, q34-q35, q21.3-q22.1, q22.2-q31.33, q21.2-q21.3, p21.2-p21.1, q36.2-q36.3, p15.2-p14.3, q31.33-q33, p12.1, p22.3-p22.2, p15.3-p15.2, q36.1, q11.21, q11.22 |
| 8 | q21.3-q24.11, q24.11-q24.3 | q22.3-q24.3 |
| 9 | q33.2 |  |
| 10 |  | p15.3-p15.1, q22.1-q22.2, q22.2-q22.3-q23.1, p13-p12.31, q11.21-q11.22, p12.1-p11.21, p12.2-p12.1, q21.3, q11.23-q21.3 |
| 11 | q21.1-q22.3, q24.3-q25, p15.2-p15.1, p15.4-p15.2, q21, q13.5-q14.1, q12.1, q24.1, q23.1, q14.3-q21, q14.1-q14.2, p11.2, p13, q23.3, p12 | q23.3 |
| 12 | q23.3-q24.11 |  |
| 13 | q12.13-q12.2 |  |
| 14 | q12, q21.2-q21.3, q12-q13.1, q13.2, q13.3-q21.2, q11.2, q32.33, q11.2 | q11.2, q32.33 |
| 15 | q11.2, q26.3, q23-q24.1, q15.1-q21.1, q11.2-q12, q21.1-q21.3, q13.1, q12-q13.1, q21.3-q22.31, q25.3-q26.1, q26.2, q25.2-q25.3, q13.1-q13.3, q24.1-q24.3, q22.31-q23, q25.1-q25.2, q13.3-q15.1 | q13.1-q13.2, q22.31-q23, q21.1, q26.2-q26.3, q13.3, q25.3 |
| 16 | q24.3, q23.2-q23.3, q24.1-q24.3, q22.3-q23.1 |  |
| 17 | q22-q23.1, q24.3-q25.1, q25.2-q25.3, q21.31-q21.32, q23.1-q24.2, q21.33-q22 | q23.2, q11.2, q21.31-q21.33, q23.1, q25.3, q24.2 |
| 19 | q12-q13.11, q13.12-q13.2, q13.42-q13.43, q13.2, q13.32-q13.33, q13.2-q13.32, q11-q12 |  |
| 20 | p13-q13.33 | q12-q13.33, p11.21, q11.21-q12 |
| 21 |  | q22.3 |
| 22 | q12.3, q13.31, q12.3-q13.2, q11.21-q12.2, q13.2-q13.31, q11.21, q11.1, q12.2-q12.3, q13.33 |  |
